# Study on Conditional Conservatism Within Fair Value Measurements Based on Anti-discount Expectations

**DOI:** 10.3389/fpsyg.2022.923055

**Published:** 2022-07-05

**Authors:** Qianlong Yu, Jiali Guo, Lili Ding, Qin Hu

**Affiliations:** ^1^Business School, University of Shanghai for Science and Technology, Shanghai, China; ^2^Industrial and Commercial Bank of China Shanghai, Shanghai, China

**Keywords:** fair value hierarchy, conditional conservatism, operating cash holdings, audit quality, internal control deficiency, discounting expectations

## Abstract

As a rapidly growing emerging capital market, China has attracted the attention of both investors and scholars. To alleviate the expectation of external users of listed companies’ financial statements to “discount” items in levels 2 and 3 of the fair value measurement, listed companies will treat these items as conditional conservatism. It refers to the conservatism of companies when confirming bad news of unrealized gains and losses sooner than confirming good news. A sample was selected for empirical analysis to verify the existence of this relationship. The results are as follows: (1) the higher the proportion of levels 2 and 3 fair value measurements, the stronger the conditional conservatism of the company’s profit and loss; (2) the higher the proportion of cash holding of operating activities in a company’s operating profit and the higher the audit quality, the higher the proportion of levels 2 and 3 fair value hierarchy measurements, and the stronger the conditional conservatism of the company’s profit and loss; and (3) the lesser the internal control defects in a company, the higher the proportion of levels 2 and 3 fair value hierarchy measurements, and the stronger the conditional conservatism of its profit and loss. The findings provide empirical evidence to identify listed companies adopting conditional conservatism to alleviate discounting expectations of fair value items of financial statements’ external users and provide a reference for improving the standards and regulation of listed companies.

## Introduction

Fair value information has played a role in fueling the global financial crisis of 2008, with many attributing its causes to fair value measurement. Al-Khadash and Khasawneh (2014) found that the quality of fair value information reflects substantial discounts during financial crises. Although the fair value measurement may have contributed to the financial crisis, it is not the root cause (Liu, 2009). The Statement of Financial Accounting Standards 157 (SFAS157), implemented by the Financial Accounting Standards Board (FASB) in 2006, is considered to be an older accounting standard for fair value measurement. The Accounting Standards for Enterprises No. 39—Fair Value Measurement (CAS39), issued by China’s Ministry of Finance in 2014, provide three fair value measurement and disclosure standards. The biased risk of fair value measurement information mainly comes from the unobservable input value information of level 3 and some approximate input values of level 2. The valuation methods of the items at levels 2 and 3 present the possibility for biased valuation behavior due to various motivations. According to Black et al. (2018), investors expect sound accounting practices without verification when faced with an uncertain economic environment. As an effective governance tool, conditional conservatism can correct biased financial reporting and create a spontaneous effective contract mechanism (LaFond and Watts, 2008; Lara et al., 2020; Garcia Osma et al., 2022; Hang et al., 2022). It refers to the conservatism of companies when confirming bad news of unrealized gains and losses sooner than confirming good news. Conditional conservatism can mitigate investors’ and other external users’ concerns about information verifiability in the case of unverifiable fair value estimates. Thus, it reduces their expectations of discounting fair value measurement items.

The overall aim of this study is to verify the levels 2 and 3 fair value hierarchy measurements of Chinese listed companies. They have a conditional conservatism effect on their profits and losses to alleviate expectations of discounting fair value measurement items and compare the differences in the influence of conditional conservatism in the group of operating cash flow proportion, audit quality, and internal control quality in operating profit. This study performs a panel regression with the extended Basu (1997) model and uses data covering 2010–2019 from listed companies in China. Our study makes a significant contribution to the literature as scholars have rarely examined the existence of conditional conservatism in the fair value stratification measurement of listed companies in emerging markets, especially the Chinese market. Further, the scholarly research on fair value measurement and the related internal and external supervisory mechanisms in China is primarily qualitative, lacking empirical studies on the impact of multi-industry, multi-period, and multi-regulatory elements of fair value information.

## Theoretical Background and Hypotheses

### Reasons for “Discounting” by External Users of Financial Statements of Fair Value Hierarchy Information

According to Barron et al. (2016), level 3 fair value disclosures could provide useful information for analysts. Ayres et al. (2017) identified that analysts’ prediction accuracy is positively correlated with the proportion of levels 2 and 3 fair value disclosures. Several scholars believe that level 2 and 3 fair value measurements lack verifiability since they rely on management assumptions (Kolev, 2008; Ramanna and Watts, 2008; Khalil et al., 2021). Further, Riedl and Serafeim (2011) asserted that companies with more financial assets that measured fair value at a lower level would have a higher risk. Sun (2017) found that fair value assets measured at levels 2 and 3 significantly positively impact bank systemic risk. As Hao and Zhang (2018) pointed out, a company’s adoption of a big data strategy can substantially enhance the value relevance of items at levels 1 and 2 fair value measurements but has no significant impact on items at level 3. Garcia Osma et al. (2022) argued that conditional conservatism acts as a mechanism that lends credibility to voluntary disclosure by providing a challenging reporting benchmark that allows outsiders to evaluate the truthfulness of management forecasts better. Due to managers’ poor verifiability and discretion, the levels 2 and 3 items of fair value measurements lead to prediction errors and decision-making risks for the external information users. Therefore, there is an expectation that the lower levels of fair value information will be discounted.

#### Conditional Conservatism

In the case of conservatism accounting, empirical evidence suggests that the confirmation of loss occurs sooner than that of income (Ball et al., 1997; Mohsin et al., 2021). Barker and McGeachin (2015) found that the International Financial Reporting Standards (IFRS) requirements can lead to the emergence of accounting conservatism, which acts as a governance mechanism. Accounting conservatism can be divided into unconditional and conditional conservatism. Beaver and Ryan (2005) stated that unconditional conservatism is also known as balance sheet conservatism or prior conservatism. Basu (1997) mentioned that conditional conservatism is also known as earnings or ex-post conservatism, implying that the confirmation of “good news” requires more conservative evidence than bad news.

Further, Cao et al. (2017) concluded that fair value deviates from unconditional conservatism but is consistent with conditional conservatism. Roychowdhury (2010) emphasized the internal and external governance mechanisms that encourage companies to increase the intensity and persistence of conditional conservatism in reports to gain benefits. As long as the incentive mechanism is sufficiently strong and continuous, a company’s annual report will include a “credible promise” for conditional conservatism (Zhang, 2008). Guo (2013) determined that the application of fair value accounting in China is consistent with the principle of accounting conservatism. Khalifa et al. (2018) found that conditional conservatism increases with the company’s debt level; when faced with higher audit litigation risks, only low-tech companies show higher conditional conservatism. Badia et al. (2017) found that in the United States securities market, the greater the financial instruments held by a company, measured at levels 2 and 3, the higher the conditional conservatism reported in its comprehensive income. Black et al. (2018) concluded that, in the financial sector of the United States securities market, conditional profit and loss conservatism are positively correlated with the proportion of levels 2 and 3 assets measured by fair value while independent of the proportion of level 1 assets. Zhang (2020) finds that greater the degree of conditional conservatism leads to lower insolvency probability, and it is better for the financial strength rating. Therefore, conditional conservatism causes contract reactions and changes in the behavior of stakeholders, thereby making accounting information more useful. When faced with uncertainty, investors expect accounting treatment under conditional conservatism. However, scholars have rarely examined the existence of conditional conservatism in the fair value hierarchy measurements of listed companies in emerging markets, especially the Chinese market.

#### Influence of Corporate Governance Mechanism on the Decision Risk of Fair Value Hierarchy Information

According to Song et al. (2010) effective corporate governance mechanisms can alleviate the information asymmetry caused by lower-level input values. Mao et al. (2014) identified that high-quality corporate governance mechanisms could reduce the risk of asset information measured at fair values. Ran (2016) considered that a higher level of corporate governance could effectively improve the quality of information disclosure related to fair value. Zeng and Wang (2016) determined that a good internal and external information environment can reduce the information risk of companies measuring assets at fair value. Gao (2016) concluded that external audits could help to curb the adverse effects of the three levels of fair value measurement items on a bank’s systemic risk spillover effects. Bens et al. (2016) found that companies improved the quality of their asset information for levels 2 and 3 after receiving a fair value-related supervision letter from the China Securities Regulatory Commission. Sun (2017) and Hsu (2017) found that companies with more level 3 fair value measurement items have higher credit risk, but this relationship weakens when they have a higher level of corporate governance. Thesing and Velte (2021) found that lower-level fair value measurements decrease earnings quality and corporate governance measures enhance earnings quality. Hence, the reliability of fair value information requires a dual guarantee mechanism for the system and technology. A high-quality internal and external supervision mechanism reduces the risk of information bias caused by levels 2 and 3 fair value measurement items. Scholarly research on fair value measurements and related internal and external supervisory mechanisms in China is predominantly qualitative, lacking empirical studies on the impact of multi-industry, multi-period, and multi-regulatory elements of fair value information.

### Hypothesis Development

As the fair value measurements at levels 2 and 3 are relatively difficult to observe and verify, the management has more opportunities for accounting manipulation. Investors and other external users of financial statements demand conditional conservatism for unverifiable fair value measurement items disclosed by companies. Conditional conservatism deals with the moral hazards due to asymmetric information generated by various aspects of the company and the inability to obtain private information from more informed parties. Conditional conservatism restricts the opportunistic behavior of the management (Watts, 2003). LaFond and Watts (2008) argued that the asymmetry of transmitted information leads to the demand for conditional conservatism, weakening managers’ ability to exaggerate financial performance and mislead investors or other external users of financial statements. Since investors and other external users of financial statements often need fair value information for decision-making. The demand for conditional conservatism increases as the verifiability of these items decreases. Companies with greater fair value measurements for levels 2 and 3 items are given more “valuation discounts” by external users of financial statements such as investors. The company will practice conditional conservatism accounting to reduce the valuation discounts on levels 2 and 3 fair value measurements. It will reduce these “valuation discounts” by dealing with unrealized losses sooner than unrealized gains.

In contrast, level 1 fair value measurement items are easily validated. The demand for conditional conservatism should be minimal for a company with more level 1 items. Accordingly, the conditional conservatism of profit and loss increases for companies with many unverifiable fair value measurements for levels 2 and 3 items. Therefore, the following hypothesis is proposed:

*H1*: The higher the proportion of items at levels 2 and 3 of fair value measurements will strengthen the conditional conservatism of the company’s profit and loss.

## Materials and Methods

### Sample Selection and Data Sources

Although CAS39 was officially implemented in July 2014, many companies had disclosed information on fair value hierarchical measurements before this point, which enabled us to collect data from 2010 to 2019. Preliminary analysis of the fair value hierarchy measurements revealed that the data disclosed by listed companies from various Shanghai and Shenzhen stock markets sectors were relatively standardized and complete. Therefore, the primary sample consisted of Shanghai A-share and Shenzhen A-share listed companies. Specifically, the fair value hierarchical information was manually extracted from the annual reports of the sample companies published on the Juchao Information Network and Oriental Fortune Network. At the same time, other data were obtained from the China Stock Market & Accounting Research Database (CSMAR) and China Center for Economic Research (CCER) databases, and the Wind database. By excluding observations with obvious irregularities or missing values, the total sample size was 2,252. The continuous variables in the model were winsorized at 2%. The empirical analysis was conducted using STATA 15.

### Regression Model and Variable Measurement

An extended Basu (1997) model was used to determine the impact of fair value hierarchical measurements information on the conditional conservatism of companies’ profits and losses. We used Basu’s approach because non-news-based measures do not capture conditional conservatism as directly as news-based measures, and returns constitute the best available summary news measure. External parties could verify firms’ fair value measurements instrument (Ryan, 2006; Badia et al., 2017). The model is shown in the following equation:


(1) CIit=β0+β1Dit+β2Retit+β3Dit×Retit+β4MTBit+β5Dit×MTBit+β6Retit×MTBit+β7Dit×MTBit×Retit+β8Lev1it+β9Dit×Lev1it+β10Retit×Lev1it+β11Dit×Lev1it×Retit+β12Lev23it+β13Dit×Lev23it+β14Retit×Lev23it+β15Dit×Lev23it×Retit+Controlsit+εit

The definitions of the variables in equation (1) are shown in Table 1. Considering the small accounting discretion of the level 1 fair value measurements, we can expect the coefficient on D*Ret*Lev1 in the above equation to be insignificant. This means that Lev1 has no significant effect on the CI, regardless of good or bad news. The report preparer is expected to perform a conditional conservatism treatment to reduce the discount evaluation of levels 2 and 3 items of fair value measurements by external users of financial statements such as investors. Hence, β_15_ on D*Lev23*Ret is expected to be positive and significant. The market-to-book (MTB) value ratio captures the degree of slack established by companies to absorb various types of “bad news” before impairment starts (Beaver and Ryan, 2005). Suppose the recoverable amount of an asset item is significantly lower than its book value. In that case, it can be realized through various impairment provisions or gains and losses from changes in fair value. Lawrence et al. (2013) regarded MTB as a proxy variable for unconditional conservatism. Therefore, equation (1) includes the D*MTB*Ret term and other control variables that affect the results.

**Table 1:** Variable definitions.

Code	Variable type	Definition
CI	Explained variable	Corporate profit and loss: operating profit divided by the total market value of equity at the beginning of the year
Ret	Explanatory variable	Average annual returns, calculated as average monthly returns over 12 months
D	Explanatory variable	If Ret <0, the key indicator variable takes the value of 1, which means “bad news,” and 0 otherwise
MTB	Explanatory variable	Market-to-book ratio: total market value divided by total book value
Lev_A1	Explanatory variable	Level 1 assets measured at fair value divided by total assets
Lev_A23	Explanatory variable	Levels 2 and 3 assets measured at fair value divided by total assets
Lev_L1	Explanatory variable	Level 1 liabilities measured at fair value divided by total assets
Lev_L23	Explanatory variable	Level 2 and 3 liabilities measured at fair value divided by total assets
Lev_AL1	Explanatory variable	The total of Level 1 assets and liabilities measured at fair value divided by total assets
Lev_AL23	Explanatory variable	The total of Level 2 and 3 assets and liabilities measured at fair value divided by the total assets
Opcas	Control variable	Average operating cash flow in profit = (average net cash flow from operating activities)/(average operating profit) × 1000
Big14	Control variable	Whether it is audited by the top 14 accounting firms in China; takes the value of 1 if audited by the top 14 accounting firms, and 0 otherwise
Isdef	Control variable	Internal control defect; takes the value of 1 if there is an internal control defect, and 0 otherwise
EquiN	Control variable	Data on the nature of equity divided into state- and non-state-owned; takes the value of 1 for a state-owned holding firm, and 0 otherwise
LEV	Control variable	Asset-liability ratio: total liabilities divided by total assets
Tover	Control variable	Annual average turnover rate, calculated as the daily average turnover rate calculated based on the number of outstanding shares during the year
Longt	Control variable	Proportion of long-term equity investment: long-term equity investment divided by owner’s equity
PSRD	Control variable	Market-to-sales ratio/1000
PERD	Control variable	P/E ratio/1000
Top10	Control variable	Shareholding ratio of the top 10 shareholders
Totin	Control variable	Institutional investor shareholding ratio
Finan	Control variable	Takes the value of 1 for the financial industry, and 0 otherwise

A two-way fixed-effects model of firm ID and year was adopted, and the robust standard deviation of the clusters was reported in this study. Table 1 shows the breakdown of the variables.

## Results

### Descriptive Statistics and Correlation Analysis

The descriptive statistics of the variables are shown in Table 2. The average disclosed proportion of the level 1 assets in the total assets measured at fair value (Lev_A1) was 0.035, representing the largest proportion of assets and liabilities measured by fair value. The average disclosure of levels 2 and 3 assets measured at fair value (Lev_A23) was 0.0344, indicating that the sampled companies disclosed fewer levels 2 and 3 assets measured by fair value. The average values of the proportion of liabilities at level 1, as well as 2 and 3 (Lev_L1 and Lev_L23) fair value measurements, were 0.0022 and 0.0018, respectively, significantly smaller than the orders of asset magnitude in the fair value measurements; both the indicators were 0 at the 75% quantile. Therefore, Lev_L1 and Lev_L23 were combined into Lev_A1 and Lev_A23, used in the following analysis. The Minimum value of annual average return rate (Ret) was negative at −0.0489; a quarter of the Ret was −0.0129, while the median was positive, indicating a positive or negative value of the annual average stock return rate for good and bad accounting news, respectively.

**Table 2:** Descriptive statistics.

Variable	Mean	SD	P25	P50	P75	Min	Max
CI	0.0244	0.0251	0.0083	0.0165	0.0332	0.0000	0.2658
Ret	0.0099	0.0309	−0.0129	0.0067	0.0301	−0.0489	0.0959
D	0.4138	0.4926	0.0000	0.0000	1.0000	0.0000	1.0000
MTB	2.2496	1.4894	1.1882	1.8043	2.8450	0.5611	9.4220
Lev_A1	0.0350	0.0754	0.0004	0.0054	0.0315	0.0000	0.7318
Lev_A23	0.0344	0.0832	0.0000	0.0000	0.0234	0.0000	0.9575
Lev_L1	0.0022	0.0165	0.0000	0.0000	0.0000	0.0000	0.3787
Lev_L23	0.0018	0.0138	0.0000	0.0000	0.0000	0.0000	0.4371
Lev_AL1	0.0373	0.0784	0.0005	0.0061	0.0348	0.0000	0.6950
Lev_AL23	0.0360	0.0860	0.0000	0.0000	0.0246	0.0000	0.7762
Opcas	0.0005	0.0565	−0.0004	0.0003	0.0015	−2.0731	1.0855
Big14	0.7230	0.4476	0.0000	1.0000	1.0000	0.0000	1.0000
Isdef	0.5211	0.4997	0.0000	1.0000	1.0000	0.0000	1.0000
EquiN	0.6196	0.4856	0.0000	1.0000	1.0000	0.0000	1.0000
LEV	0.5254	0.1994	0.3802	0.5304	0.6728	0.0188	0.9659
PSRD	0.0045	0.0144	0.0009	0.0019	0.0039	0.0001	0.3393
PERD	0.0477	0.0972	0.0131	0.0225	0.0446	0.0021	1.4539
Tover	1.6661	1.4025	0.6945	1.2390	2.2291	0.0418	10.1344
Longt	0.3813	0.2486	0.1688	0.3547	0.5584	0.0003	1.0000
Top10	0.5319	0.1796	0.4133	0.5341	0.6554	0.0012	0.9614
Totin	0.4752	0.2514	0.2886	0.5003	0.6714	0.0000	0.9819
Finan	0.0812	0.2732	0.0000	0.0000	0.0000	0.0000	1.0000

### Correlation Analysis

The results of the Pearson correlation test are presented in Table 3. The proportion of levels 2 and 3 items measured by fair value (Lev_AL23) was significantly positively correlated with operating profit and annual average return. In contrast, the proportion of level 1 items measured by fair value (Lev_AL1) was significantly positively correlated with annual average returns. There was no significant collinearity among the variables. The results of the Spearman’s correlation test did not differ significantly from those of the Pearson’s correlation test.

**Table 3:** Correlation analysis.

	CI	Ret	D	MTB	Lev_AL1	Lev_AL23	Opcas	Big14	Isdef	EquiN	LEV	PSRD	PERD	Tover	Longt	Top10r	Totin	Finan
CI	1																	
Ret	0.17^**^	1																
D	−0.15^**^	−0.85^**^	1															
MTB	0.00	0.32^**^	−0.23^**^	1														
Lev_AL1	0.03	−0.05^*^	0.06^**^	0.06^**^	1													
Lev_AL23	0.06^**^	0.07^**^	−0.08^**^	−0.24^**^	−0.13^**^	1												
Opcas	0.02	0.04^*^	−0.05^*^	−0.03	−0.13^**^	0.02	1											
Big14	−0.02	−0.06^**^	0.07^**^	0.01	−0.07^**^	0.03	0.04	1										
Isdef	−0.10^**^	−0.12^**^	0.10^**^	−0.07^**^	0.04	0.06^**^	−0.04^*^	−0.03	1									
EquiN	−0.02	−0.09^**^	0.11^**^	−0.06^**^	0.02	−0.20^**^	0.05^*^	0.10^**^	0.06^**^	1								
LEV	−0.26^**^	0.02	0.01	−0.29^**^	−0.16^**^	0.24^**^	0.03	0.15^**^	−0.00	0.00	1							
PSRD	0.06^**^	0.13^**^	−0.06^**^	0.50^**^	0.20^**^	0.06^**^	−0.10^**^	−0.02	−0.05^*^	−0.09^**^	−0.36^**^	1						
PERD	−0.38^**^	0.11^**^	−0.05^**^	0.57^**^	0.10^**^	−0.24^**^	−0.03	−0.06^**^	0.01	−0.00	−0.28^**^	0.38^**^	1					
Tover	−0.12^**^	0.34^**^	−0.25^**^	0.43^**^	0.06^**^	−0.17^**^	−0.04^*^	−0.05^*^	−0.06^**^	−0.06^**^	−0.09^**^	0.19^**^	0.39^**^	1				
Longt	−0.13^**^	−0.04	0.02	−0.01	0.03	−0.14^**^	−0.18^**^	−0.04	0.04^*^	−0.07^**^	−0.12^**^	−0.06^**^	0.03	0.03	1			
Top10r	0.12^**^	0.02	−0.02	−0.15^**^	−0.10^**^	0.07^**^	0.05^*^	0.12^**^	0.06^**^	0.18^**^	0.10^**^	−0.14^**^	−0.22^**^	−0.49^**^	−0.03	1		
Totin	0.06^**^	−0.28^**^	0.21^**^	−0.25^**^	−0.05^**^	0.19^**^	−0.00	0.03	0.13^**^	0.05^*^	0.09^**^	−0.11^**^	−0.25^**^	−0.45^**^	0.05^*^	0.44^**^	1	
Finan	−0.07^**^	−0.04	0.05^*^	−0.10^**^	0.17^**^	0.42^**^	−0.03	0.06^**^	0.04	−0.09^**^	0.33^**^	0.32^**^	−0.12^**^	−0.06^**^	−0.26^**^	−0.05^*^	0.06^**^	1

*p < 0.1; **p < 0.05.

### Regression and Robustness Analyses

#### Regression Analysis

The regression results of equation (1) are shown in the CI1 column of Table 4. The coefficient on D*Lev_AL23*Ret for corporate operating profit (CI) was 1.7772, positively significant and consistent with our expectations. This implied that companies with higher fair value measurements for levels 2 and 3 would report bad news more promptly and treat good news cautiously to alleviate the expectation that fair value measurement items would be discounted. Therefore, such companies exhibit significant conditional conservatism, verifying H1. The coefficient on D*Lev_AL1*Ret was 0.1409, insignificant and consistent with our expectations. The level 1 items measured at fair value are easy to verify, and such items do not impact the conditional conservatism of CI.

**Table 4:** Regression results.

Variables	CI1	CI2	CI3
D	−0.0033	−0.0033	−0.0039
	(0.0031)	(0.0031)	(0.0031)
Ret	0.0047	0.0032	−0.0035
	(0.0525)	(0.0522)	(0.0516)
D*Ret	0.1284	0.1286	0.1300
	(0.1259)	(0.1255)	(0.1236)
MTB	0.0005	0.0005	0.0005
	(0.0012)	(0.0012)	(0.0012)
D*MTB	0.0012	0.0012	0.0011
	(0.0011)	(0.0011)	(0.0011)
Ret*MTB	0.0073	0.0073	0.0065
	(0.0129)	(0.0129)	(0.0129)
D*MTB*Ret	0.0271	0.0283	0.0297
	(0.0412)	(0.0414)	(0.0411)
Lev_AL1	−0.0017		
	(0.0189)		
D*Lev_AL1	−0.0216		
	(0.0206)		
Ret*Lev_AL1	−0.3168		
	(0.3410)		
D*Lev_AL1*Ret	0.1409		
	(0.6175)		
Lev_AL23	−0.0111		−0.0088
	(0.0157)		(0.0153)
Ret*Lev_AL23	0.1059		0.1127
	(0.1549)		(0.1535)
D*Lev_AL23	0.0399		0.0379
	(0.0243)		(0.0237)
D*Lev_AL23*Ret	1.7772^*^		1.6919^*^
	(0.9485)		(0.9430)
Lev_A1		0.0042	
		(0.0190)	
D*Lev_A1		−0.0233	
		(0.0219)	
Ret*Lev_A1		−0.3155	
		(0.3528)	
D*Lev_A1*Ret		0.1155	
		(0.6600)	
Lev_A23		−0.0127	
		(0.0176)	
D*Lev_A23		0.0413	
		(0.0277)	
Ret*Lev_A23		0.1235	
		(0.1734)	
D*Lev_A23*Ret		1.9077^*^	
		(1.0574)	
Opcas	−0.0911	−0.0890	−0.0908
	(0.0794)	(0.0788)	(0.0786)
Big14	−0.0012	−0.0012	−0.0011
	(0.0022)	(0.0022)	(0.0022)
Isdef	−0.0029^**^	−0.0029^**^	−0.0029^**^
	(0.0015)	(0.0015)	(0.0015)
LEV	−0.0552^***^	−0.0550^***^	−0.0542^***^
	(0.0125)	(0.0126)	(0.0125)
Top10	−0.0168^***^	−0.0168^***^	−0.0167^***^
	(0.0058)	(0.0058)	(0.0058)
Controls	YES	YES	YES
Constant	0.0753^***^	0.0750^***^	0.0739^***^
	(0.0115)	(0.0116)	(0.0113)
Observations	2,252	2,252	2,252
*R*-squared	0.1160	0.1158	0.1147
ID FE	YES	YES	YES
Year FE	YES	YES	YES

Clustered Robust SEs are shown in parentheses.

*** *p* < 0.01;

** *p* < 0.05;

* *p* < 0.1;

The debt-to-asset ratio had a significant negative impact on CI, indicating that, with an increase in the debt-to-asset ratio (LEV), the company’s debt servicing cost increases, which erodes its profit to a certain extent. The profitability of a company ultimately depends on the profitability of its products. Firms without internal control defects (Isdef) had higher returns than those with internal control defects. The higher the shareholding ratio (Top10) of the top 10 shareholders, the lower the CI. There is a possibility that China’s concentration of equity has aggravated the infringement of large shareholders on the interests of small shareholders and corporate interests and reduced CI.

#### Robustness Analysis

Since assets and liabilities measured by fair value have different benefits for CI and because Lev_L1 and Lev_L23 are still 0 at the 75% quantile in Table 2, most liabilities measured by fair value were almost 0. After deducting the impact of the liabilities measured by fair value, Lev_AL1 and Lev_AL23 were replaced with Lev_A1 and Lev_A23 for levels 1, 2, and 3 fair value assets measurements, respectively, and introduced into equation (1) to validate H1. The results are shown in column CI2 of Table 4. The coefficient on D*Lev_AL23*Ret was significant at 1.9077, supporting H1.

As shown in Table 3, there is a certain correlation between the items at levels 1, 2, and 3 fair value measurements, with the correlation coefficient as −0.13 (significant at the 0.01 level). Therefore, Lev_AL1 was removed from the equation (1), while the other variables were retained to verify the validity of H1. The results are shown in column CI3 of Table 4. The coefficient on D*Lev_AL23*Ret was significant at 1.6919, supporting H1.

## Additional Analysis

In view of the different financial contexts of companies and their corporate governance environments, the fair value hierarchy measurement information will have varying impacts on companies’ profits and losses. Therefore, considering the proportion of operating cash holdings in operating profit (profit quality dimension), audit quality dimension, and internal control quality dimension as grouping variables, this study further analyzed the impact of conditional conservatism of fair value hierarchy measurements information on companies’ profits and losses, to alleviate the expectation that fair value measurement items will be discounted under different scenarios.

### Grouping Analysis Based on the Percentage of Operating Cash Holdings in Operating Profit

Even within the scope of accounting standards, with the exception of listed companies that deliberately violate accounting standard provisions and commit financial fraud, there will be differences in the application of accounting standards and accounting recognition, measurement, recording, and presentation. Accounting profits can be manipulated to a certain extent. Compared with accounting profits, cash (generalized cash, including cash, bank deposits, and other monetary funds) holdings exist and are less likely to be fictitious. For instance, financial personnel can inflate operating profits by inflating receivables and related operating income, but it is difficult to increase cash through such “book games.” Such firms will have a clearer “bottom line” on their fair value hierarchy measurements, resulting in a more conditional conservatism accounting treatment to alleviate the expectation that fair value measurement items will be discounted. Thus, we propose the following hypothesis:

*H2*: The higher the proportion of a company’s cash holdings of operating activities in operating profit, the higher the proportion of levels 2 and 3 items fair value measurements, and the stronger the conditional conservatism of its profits and losses.

We examined the impact of fair value hierarchy measurements on the conditional conservatism of companies’ profits and losses under different scenarios using Opcas as a grouping variable that indicates average cash holdings of operating activities in the operating profit. As shown in Table 5, Opcas tests the validity of H2 under different grouping environments by using the median P50 (0.0003) and average mean (0.0005) as the grouping basis.

**Table 5:** Groups based on the percentage of operating cash holdings.

Variables	H_Opcas_P50	L_Opcas_P50	H_Opcas_Mean	L_Opcas_Mean
D	−0.0021	−0.0005	−0.0061	−0.0022
	(0.0048)	(0.0054)	(0.0047)	(0.0053)
Ret	0.1105	−0.0247	0.0731	−0.0289
	(0.0819)	(0.0979)	(0.0899)	(0.0922)
D*Ret	−0.2521	0.4922^**^	−0.3363^*^	0.4568^**^
	(0.1945)	(0.2038)	(0.1892)	(0.1938)
MTB	0.0011	−0.0021	0.0006	−0.0012
	(0.0015)	(0.0021)	(0.0015)	(0.0020)
D*MTB	0.0019	0.0005	0.0034^**^	0.0007
	(0.0016)	(0.0018)	(0.0017)	(0.0017)
Ret*MTB	−0.0102	0.0122	0.0093	0.0171
	(0.0197)	(0.0261)	(0.0203)	(0.0233)
D*MTB*Ret	0.1513^**^	−0.0464	0.1621^**^	−0.0483
	(0.0671)	(0.0553)	(0.0688)	(0.0548)
Lev_AL1	−0.0262	−0.0028	−0.0491	0.0065
	(0.0320)	(0.0281)	(0.0385)	(0.0238)
D*Lev_AL1	−0.0019	−0.0183	0.0115	−0.0173
	(0.0470)	(0.0247)	(0.0485)	(0.0245)
Ret*Lev_AL1	0.0238	0.2179	0.0684	0.0804
	(0.4584)	(0.5903)	(0.5056)	(0.5622)
D*Lev_AL1*Ret	0.3058	−0.3923	0.6887	−0.2878
	(1.8070)	(0.7441)	(1.8097)	(0.7094)
Lev_AL23	−0.0166	−0.0072	−0.0018	−0.0037
	(0.0293)	(0.0219)	(0.0345)	(0.0203)
Ret*Lev_AL23	−0.0316	0.0107	−0.0456	0.0119
	(0.0636)	(0.0346)	(0.0705)	(0.0338)
D*Lev_AL23	−0.7217	0.0446	−1.0990	0.0473
	(0.9377)	(0.1851)	(1.1068)	(0.1853)
D*Lev_AL23*Ret	2.0656^*^	0.8929	2.3900^*^	0.4847
	(1.2381)	(1.5267)	(1.3306)	(1.3601)
Controls	YES	YES	YES	YES
Constant	0.0446^***^	0.0532^***^	0.0452^***^	0.0511^***^
	(0.0127)	(0.0123)	(0.0138)	(0.0114)
Observations	1,117	1,135	1,010	1,242
R-squared	0.1321	0.1034	0.1420	0.0950
ID FE	Yes	Yes	Yes	Yes
Year FE	Yes	Yes	Yes	Yes

Clustered Robust SEs are shown in parentheses.

*** *p* < 0.01;

** *p* < 0.05;

* *p* < 0.1.

Columns (2) and (3) of Table 5 show the regression results of the median (P50) of operating cash holdings in the operating profit index (Opcas). The coefficient on D*Lev_AL23*Ret was significant in the high median group of Opcas (H_Opcas_P50), at 2.0656, while the coefficient on D*Lev_AL23*Ret was not significant in the low Opcas median group (L_Opcas_P50), at 0.8929. Columns (4) and (5) of Table 5 show the regression results grouped by the mean of Opcas. The coefficient on D*Lev_AL23*Ret was significant in the high group (H_Opcas_Mean) of the mean of Opcas, at 2.39, while it was not significant in the low group (L_Opcas_Mean), at 0.4847. Therefore, when the proportion of a company’s cash holdings of operating activities in the operating profits is relatively high, and if the proportion of items at levels 2 and 3 fair value hierarchy measurements is higher, the conditional conservatism of its profit and loss is stronger. In other words, H2 holds. This is primarily because as the cash holdings of operating activities account for a relatively high proportion of operating profits, companies are more confident in managing unrealized gains and losses under accounting conservatism; they deal with bad news in a timelier manner than the good news.

### Comparative Analysis Based on Audit Quality Grouping

According to DeFond et al. (2016), independent third-party auditors charge lower audit fees for sound clients, reduce concerns about the company’s sustainability, and work with the company for longer periods of time, suggesting that auditors appreciate if clients adopt conditional conditions conservatism. Auditors with good reputations and potential litigation problems are more likely to require conservatism (Basu et al., 2001; Chung et al., 2003). A high-quality audit often provides better external supervision to the audited company; in this case, the audited company will provide more reliable information, including level 2 and 3 fair value measurements for conditional conservatism. Besides the company’s direct handling of conditional conservatism at level 2 and 3 fair value measurements, employing high-quality auditors is an indirect requirement of external stakeholders, such as investors. The satisfaction mechanism can alleviate the concerns of investors and other external report users regarding unverifiable information. Thus, we propose the following hypothesis:

*H3*: When the audit of a company is of high quality and the proportion of the items at level 2 and 3 fair value measurements is higher, the conditional conservatism in their profits and losses will be stronger.

The Chinese Institute of Certified Public Accountants annually publishes the “List of Top 100 Domestic Accounting Firms (Comprehensive Evaluation),” based on which the audit services provided by the top 14 accounting firms for the sample companies during 2010–2019 were selected as a proxy variable for high-quality audit (Big14). The audit services provided by accounting firms that are not on this list for the sample companies are considered in the low-quality audit group. Considering the Big14 as a grouping variable, this study further examines the impact of fair value hierarchy measurements on the conditional conservatism of companies’ profits and losses under different scenarios of conditional conservatism. It tests the validity of H3 under different grouping environments, as shown in Table 6.

**Table 6:** Regression results under audit quality grouping.

Variables	Big14	Non_Big14
D	−0.0010	−0.0164
	(0.0033)	(0.0108)
Ret	0.0175	−0.1192
	(0.0525)	(0.1731)
D*Ret	0.0953	0.2146
	(0.1370)	(0.3762)
MTB	0.0015	−0.0027
	(0.0012)	(0.0047)
D*MTB	(0.0134)	(0.0440)
	0.0006	0.0034
Ret*MTB	−0.0018	0.0507
	(0.0013)	(0.0034)
D*MTB*Ret	0.0485	0.0005
	(0.0459)	(0.0959)
Lev_AL1	−0.0325	0.0456
	(0.0258)	(0.0308)
D*Lev_AL1	−0.0433^*^	0.0161
	(0.0256)	(0.0315)
Ret*Lev_AL1	−0.1629	−0.3386
	(0.4399)	(0.5675)
D*Lev_AL1*Ret	−1.3975^**^	1.6823^**^
	(0.6741)	(0.8454)
Lev_AL23	−0.0247	−0.0089
	(0.0163)	(0.0373)
D*Lev_AL23	0.0525^*^	0.0319
	(0.0268)	(0.0556)
Ret*Lev_AL23	0.2545^*^	0.2087
	(0.1522)	(1.0072)
D*Lev_AL23*Ret	1.7589^*^	0.4964
	(0.9909)	(2.4206)
Controls	Yes	Yes
Observations	1,626	626
R-squared	0.1153	0.1609
ID FE	Yes	Yes
Year FE	Yes	Yes

Clustered Robust SEs are shown in parentheses.

^***^p < 0.01;

** *p* < 0.05;

* *p* < 0.1.

Columns (2) and (3) of Table 6 show the regression results using Big14 as the grouping variable. The coefficient on D*Lev_AL23*Ret is significant in the high-quality audit group of Big14, at 1.7589. The coefficient on D*Lev_AL23*Ret is not significant in the low-quality audit group of Big14, at 0.4964. Therefore, when audit quality is high, and the proportion of a company’s items at level 2 and 3 fair value measurements is higher, the conditional conservatism in their profits and losses is stronger. In other words, H3 holds. In general, when reputed third-party accounting firms audit listed companies, the audit quality of these companies will improve. For their reputation, continuous operations weaker investors and other external report users’ concerns about level 2 and 3 fair value measurements. The listed companies will adopt more cautious attitudes in preparing financial statements and disclosure of financial statements, which are in line with the conservatism requirements.

### Comparative Analysis of Grouping Based on Whether There Are Internal Control Defects

The internal control of a company has a significant impact on the quality of its financial reporting information. Effective internal control can be classified as content as the effective internal control of financial and non-financial reports. Internal control exists throughout the entire process of a company’s information flow, whether it is collection and sorting or the reporting and disclosure of information and whether it pertains to financial or non-financial information. For firms with more effective internal control, especially concerning financial reporting, the design of the internal control system is relatively complete, and the implementation of the relevant system is relatively strict. Companies are more likely to be cautious for the evaluation and information disclosure of levels 2 and 3 fair value measurement-related items that require large accounting discretion. They are more likely to carry out conditional conservatism accounting when dealing with the unrealized gains and losses caused by levels 2 and 3 fair value measurements. Therefore, the following hypothesis is proposed:

*H4*: In the absence of internal control deficiencies, the higher the proportion of items at levels 2 and 3 fair value measurements, the stronger the conditional conservatism of the company’s profit and loss.

To further test the validity of H4 under different grouping environments, groups were based on an indicator (Isdef) of whether the sampled companies had internal control defects (data from the CSMAR database), as shown in Table 7.

**Table 7:** Grouping results of internal control defects.

Variables	Non_deficiency	Deficiency
D	−0.0073	−0.0039
	(0.0058)	(0.0041)
Ret	0.0105	0.0773
	(0.0864)	(0.0811)
D*Ret	0.0066	−0.0344
	(0.2557)	(0.1548)
MTB	0.0010	−0.0020
	(0.0023)	(0.0014)
D*MTB	0.0018	0.0018
	(0.0025)	(0.0014)
Ret*MTB	−0.0008	−0.0002
	(0.0208)	(0.0213)
D*MTB*Ret	0.1174	0.0602
	(0.0882)	(0.0546)
Lev_AL1	−0.0118	−0.0263
	(0.0299)	(0.0251)
D*Lev_AL1	0.0174	0.0212
	(0.0433)	(0.0265)
Ret*Lev_AL1	−0.0842	0.5784
	(0.5684)	(0.4170)
D*Lev_AL1*Ret	0.8991	−0.0703
	(1.0170)	(0.7634)
Lev_AL23	−0.0260	0.0075
	(0.0340)	(0.0228)
D*Lev_AL23	0.0836^**^	−0.0001
	(0.0418)	(0.0231)
Ret*Lev_AL23	0.3734^**^	−0.1796
	(0.1827)	(0.4523)
D*Lev_AL23*Ret	4.0340^**^	0.3682
	(1.8113)	(0.8744)
Controls	Yes	Yes
Observations	1,078	1,174
*R*-squared	0.1234	0.1158
ID FE	Yes	Yes
Year FE	Yes	Yes

Clustered Robust SEs are shown in parentheses.

^***^p < 0.01;

** *p* < 0.05;

^*^p < 0.1.

Columns (2) and (3) of Table 7 show the regression results when internal control defect (Isdef) was the grouping variable. The coefficient of D*Lev_AL23*Ret was significant in the non-existent internal control defect group (Non_deficiency column) at 4.0340. The coefficient of D*Lev_AL23*Ret was not significant in the internal control defect group (Deficiency column), at 0.3682. This shows that when a company’s internal control has defects, the proportion of levels 2 and 3 fair value measurements has no significant effect on the conditional conservatism of their profits and losses. When a company’s internal control has no defects, the higher the proportion of levels 2 and 3 items fair value measurements, the stronger the conditional conservatism in its profits and losses. When the internal control system of a company is more effective, the company will pay more attention to the accuracy and rigor of financial information disclosure, so the relevant information of levels 2 and 3 items measured by fair value will be more carefully disclosed and conditional conservatism of the company’s profit and loss will be stronger. In other words, H4 holds.

## Discussion

### Theoretical Contributions and Implications for Management

Our study makes a significant contribution to the literature. Firstly, scholars have rarely examined the existence of conditional conservatism for alleviating expectations of discounting fair value measurement items of listed companies in emerging markets, especially the Chinese market. Secondly, the scholarly research on fair value measurement and the related internal and external supervisory mechanisms in China is mostly qualitative, lacking empirical studies on the impact of multi-industry, multi-period, and multi-regulatory elements of fair value information. Lastly, the study systematically analyzes mechanisms underlying the risk of information bias in the fair value measurement of Chinese enterprises and to provide a reference for managing these risks.

Meanwhile, our study makes a significant contribution to the implications for management. On the one hand, the findings relating to conditional conservatism, identification of internal regulatory elements, and other relevant research findings will guide the application of accounting treatment and internal corporate governance for Chinese enterprises. On the other hand, the study’s findings will provide a reference for improving Chinese accounting standards and the supervision of listed companies.

### Study Limitations and Future Research Suggestions

The limitations of the study are as follows: firstly, as the accounting standards for fair value hierarchy began to be formally implemented in China in 2014, and very few listed companies made fair value hierarchy disclosures before 2010, all disclosure information on fair value hierarchy of listed companies was collected manually. Limited by the difficulty of data collection, the article collects data from 2010 to 2019, which is a short time span. Meanwhile, to avoid the negative impact of the COVID-19 pandemic on listed companies, data beyond 2020 were not collected. Finally, in the Chinese capital market in 2018, a rumor spread about the accounting treatment of goodwill being tested for impairment at year-end, which will be changed to amortization and charged to current expense. A total of 464 sample companies with non-zero goodwill were obtained by querying the Wind database to reduce the impact of this change on the findings of this paper. There are only four sample companies that match the conditions we need for regression analysis. We did not conduct further analysis due to the small amount of data, and the impact of goodwill impairment can be left for further studies.

This study can be extended in the following areas. Firstly, the investors’ expectations of discounts on fair value measurement items and the accounting treatment of the conditional conservatism of CFOs of listed companies can be investigated. The case studies could provide an in-depth analysis of the extent to which investors’ expectations of discounts to fair value impact the adoption of the conditional conservatism accounting treatment of fair value measurement items by listed companies. Secondly, further consideration can be given to other grouping scenarios; for example, a grouping of the level or type of institutional investor ownership can be included to study the conditional conservatism of the fair value hierarchy under different grouping scenarios. Finally, the research on topics such as the impact of fair value measurement items on undesired earnings and surplus management by listed companies based on fair value measurement items are all desirable.

## Conclusion

Compared with level 1 items of fair value measurements, levels 2 and 3 items of fair value measurements show higher maneuverability, which provides favorable conditions for management opportunism. Conditional conservatism has become convenient and low-cost for statement preparers (compared with paying a third-party institution to enhance their credit). A total of 2,252 listed companies were selected from China’s prosperous and emerging Shanghai A-share and Shenzhen A-share main board markets between 2010 and 2019. The impact of a fair value hierarchy measurement on a company’s profit and loss was analyzed based on conditional conservatism. Due to the differences in companies’ financial conditions and corporate governance environments, the impact of fair value hierarchical measurement information on their profits and losses will differ. Therefore, considering the proportion of operating cash holdings in the operating profit (profit quality dimension), audit quality dimension, and internal control quality dimension as grouping variables, this study examined the conditional conservatism of fair value hierarchy measurements information on a company’s profit and loss under different scenarios. The findings are as follows:

1. The higher the proportion of items at levels 2 and 3 of fair value measurements, the stronger the conditional conservatism of the company’s profit and loss.
2. The higher the proportion of cash holding of operating activities in a company’s operating profit and the higher the audit quality, the higher the proportion of levels 2 and 3 fair value hierarchy measurements and the stronger the conditional conservatism of its profit and loss.
3. The lesser the internal control defects in a company, the higher the proportion of levels 2 and 3 fair value hierarchy measurements, and the stronger the conditional conservatism of its profit and loss.

The findings provide empirical evidence to identify listed companies adopting conditional conservatism to alleviate discounting expectations of fair value items of financial statements’ external users. These conclusions serve as a reference for improving the standards and supervision of listed companies.

## Data Availability Statement

Publicly available datasets were analyzed in this study. This data can be found at: Juchao Information Network, http://www.cninfo.com.cn/new/index, Oriental Fortune Network, https://www.eastmoney.com/, China Stock Market & Accounting Research Database, https://www.gtarsc.com/, CCER Economic and Financial Database, http://www.ccerdata.cn/, and Wind Database, https://www.wind.com.cn/.

## Author Contributions

QY: conceptualization, methodology, and writing-original draft preparation. JG: investigation and validation. LD: data curation and writing-original draft preparation. QH: software and writing- original draft preparation. All authors co ed to the article and approved the submitted version.

## Funding

We acknowledge the financial supports of the Shanghai Accounting Association (SHKJ2018YB02) and Shanghai High-level Discipline Construction Project (Management Science and Engineering).

## Conflict of Interest

Author LD was employed by the company Industrial and Commercial Bank of China Shanghai.

The remaining authors declare that the research was conducted in the absence of any commercial or financial relationships that could be construed as a potential conflict of interest.

## Publisher’s Note

All claims expressed in this article are solely those of the authors and do not necessarily represent those of their affiliated organizations, or those of the publisher, the editors and the reviewers. Any product that may be evaluated in this article, or claim that may be made by its manufacturer, is not guaranteed or endorsed by the publisher.
